# 

**Published:** 1875-05

**Authors:** 


### Table of Contents.

ORIGINAL COMMUNICATIONS.

HERNIA. *By J. H. Pooley, M.D., of Yonkers, N. Y.* .................... 65

PHLAGMASIA DOLENS, PUERPERAL FEVER, AND MAMMARY ABSCESS. *By B. H. Washington, M.D.* .................... 76

NARROW ESCAPES. *By L. Alexander, M.D., of Yorkville, S. C.* .......... 79

CLINICAL STUDIES with Large Non-Emetic Doses of Ipecacuanha: With a Contribution to the Therapeusis of Cholera. *By Alfred A. Woodhull, M.D., Asst. Surg. U. S. A.* (*Concluded*) .................... 80

ATLANTA ACADEMY OF MEDICINE.

Intestinal obstruction—Distensile enema in strangulated hernia—Anal fistula, case- Fistula in consumptives—Cataract in the aged—Case trephined for epilepsy—Case, hemorrhoids—Vicarious menstruation —Epigastric tumor—Non-emetic use of ipecacuanha in intermittents —Opium poisoning —Antagonism of morphia and atropia—Ruptured perineum—Perineal support in labor—Management of puerperal Women—Removal of placenta by Crede's method .................. 94

Essay on the Actions of Mercury .................... 104

OUR STATE SOCIETIES.

Medical Association of Alabama .................... 109

Medical Association of Georgia .................... 115

OUR EXCHANGES.

Odontalgia .................... 123

Geology and the Bible .................... 124

Management of Hydrocele .................... 125

Infinitesimal Dosing .................... 126

EDITORIAL.

Association at Savannah .................... 127

Miscellaneous Notes .................... 128

